# Emotional food craving across the eating disorder spectrum: an ecological momentary assessment study

**DOI:** 10.1007/s40519-024-01690-4

**Published:** 2024-09-12

**Authors:** Ann-Kathrin Arend, Jens Blechert, Takuya Yanagida, Ulrich Voderholzer, Julia Reichenberger

**Affiliations:** 1 Department of Psychology, Centre for Cognitive Neuroscience, Paris-Lodron University of Salzburg, Salzburg, Austria; 2 School of Applied Health and Social Sciences, University of Applied Sciences Upper Austria, Linz, Austria; 3 Department of Psychiatry and Psychotherapy, University Hospital of the LMU Munich, Munich, Germany; 4 Department of Psychiatry and Psychotherapy, University Hospital Freiburg, Freiburg, Germany

**Keywords:** Food craving, Emotions, Emotional eating, Eating disorders, Ecological momentary assessment, Experience sampling method

## Abstract

**Purpose:**

Emotional eating during negative emotions might underlie disordered eating behavior (i.e., binge eating and food restriction). Positive emotions, by contrast, seem to promote healthier eating behavior. Naturalistic research on the links between emotions and eating across individuals with binge-eating disorder (BED), bulimia nervosa (BN), binge-purge anorexia nervosa (AN-BP), and restrictive anorexia nervosa (AN-R) is, however, lacking.

**Methods:**

Individuals without eating disorders (comparison group, CG, *n* = 85), and patients with BED (*n* = 41), BN (*n* = 50), AN-BP (*n* = 26), and AN-R (*n* = 29) participated in an ecological momentary assessment study. Six daily notifications over eight days prompted ratings of momentary food craving and emotional states differing in valence and arousal.

**Results:**

Results supported specific emotion-food-craving patterns in each group. Compared to the CG, arousing negative emotions and higher cravings co-occurred in patients with BN. In patients with AN-BP (at trend level also in patients with AN-R) less arousing negative emotions and lower cravings co-occurred. In patients with AN, positive emotions and higher cravings co-occurred whereas in patients with BED less arousing positive emotions and lower cravings co-occurred.

**Conclusion:**

The found emotion-craving associations may underlie group-specific (dys-)functional eating behaviors, i.e., binge eating and food restriction during negative emotions in patients with BN and AN, and normalized appetitive responses during positive emotions in patients with BED and AN. Therapeutic efforts could target arousing negative emotions in patients with BN, and less arousing negative emotions in patients with AN. Positive emotions could be used in a salutogenetic approach in patients with BED and AN.

## Introduction

Emotional eating typically refers to associations of negative emotional states with overeating that are not driven by homeostasis [1]. Overeating in connection with negative emotions is considered a risk and maintenance factor for eating disorders [2, 3], but *under*eating has been reported to be affected by negative emotions as well, while positive emotions also seem to play a role in this context [4]. Questionnaire research suggests that the different subtypes of eating disorders have specific emotional eating ‘profiles’ [e.g., 4]. The current study investigates food cravings and their associations with positive and negative emotional states in patients with binge-eating disorder (BED), bulimia nervosa (BN), the binge-purge subtype of anorexia nervosa (AN-BP), the restrictive subtype of anorexia nervosa (AN-R), and individuals without (lifetime) eating disorders (‘comparison group’, CG).

### Emotional eating theories

This section introduces theories explaining changes in eating behaviors due to emotional states. Learning-based theories such as the *affect regulation model* have been prominent in emotional eating research [5]. Since an intake of palatable foods can improve the emotional state, individuals might learn to resort to this response when in negative emotional states even in the absence of hunger. Overeating in response to negative emotional states is thus maintained through negative reinforcement [1, 5]. At higher intensities, such overeating might escalate into binge eating [2, 6, 7]. On the other hand, severe food restriction (i.e., in patients with AN) could fulfill a similar inhibition or escape function in the regulation of negative emotions [cf. affect regulation model, escape theory and emotional or experiential avoidance; 5, 8, 9]. Here, the affect regulation account would assume that weight fears are reduced through restriction, a mechanism that is also maintained through negative reinforcement.

Moreover, emotional eating is not just limited to negative emotions. Recent evidence suggests normalizations of food intake during *positive emotions* in individuals with eating disorders [4, 10–12]. The affect regulation model does not apply here since positive emotions do not elicit the need for downregulation. However, positive emotions have been shown to ‘broaden’ the cognitive-behavioral repertoire and counteract negative emotions [cf. broaden-and-build theory of positive emotions; 13–15]. Through this mechanism, positive emotions could make health goals more accessible in individuals with an eating disorder [16]. Thus, positive emotions may help patients to make better decisions about their behaviors, possibly leading to less restriction in patients with AN and less binge eating in patients with BN and BED.

### Evidence for emotional eating in different eating disorders

Questionnaire research offers some support for the aforementioned theories: In line with the affect regulation model, patients with binge-type eating disorders (BN, BED) reported overeating during negative emotions, while patients with restrictive-type eating disorders (AN) reported undereating—the opposite pattern [4, 17]. In line with the broaden-and-build theory, patients with ANr, ANbp, BN and BED reported adopting healthier eating patterns during positive emotional states [less overeating in binge-type eating disorders—less undereating in restrictive-type eating disorders; 4]. While some studies suggest that positive emotions lead to healthier eating behaviors in eating disorders, other studies indicate that they can also trigger unhealthy eating behaviors [18–21], highlighting the need for further investigation.

#### Evidence from ecological momentary assessments

To avoid reporting biases of emotional eating trait questionnaires [22] and to capture the contextualized nature of eating behavior in everyday life, ecological momentary assessments (EMA) is considered the method of choice [23]. Regarding *negative emotions,* EMA studies in patients with binge-type eating disorders (BED, BN, AN-BP) found negative emotions to precede binge eating [24–26]. Crucially, and consistent with the negative reinforcement assumption of the affect regulation model, other studies showed that negative emotions are reduced after binge-eating episodes, at least in the short term [27–30]. In patients with restrictive eating disorders (AN-R and AN-BP), negative affect was associated with a higher likelihood of restriction (i.e., undereating) at the following day [31], which is consistent with the affect regulation model.

However, not all EMA research is consistent with the described patterns and theories: While negative emotions seem to improve briefly after binge-eating episodes [29], they seem to aggravate over the longer term [27], which appears to be inconsistent with the negative reinforcement assumption of the affect regulation model. However, the more immediate reinforcing consequence may be the more potent reward mechanism and, thus in line with the affect regulation model. Further, in patients with AN, the temporal sequence (and causal direction) of negative emotions and restriction is not as clear as there are mostly day-level data that only show covariation of negative emotions and restriction [32]. In addition, some evidence was found for negative emotional overeating patterns in patients with AN who experience binge eating [31] and for negative emotional undereating in patients with binge-type eating disorder [33]. These ambiguities and the limited literature across different eating disorders highlight the need for further EMA research on emotional states and eating behaviors.

### Mapping the emotional space on valence and arousal

The type of emotions that drive emotional eating seem to differ between eating disorders, e.g., sadness might be related to undereating in patients with AN-R but not in patients with AN-BP [4]. Likewise, while binge-eating might be related to anger and frustration in patients with BN, depressiveness might play a role in patients with BED [34]. However, as different studies assessed different emotions it becomes difficult to determine overall emotional eating patterns. There is currently no consensus about which emotional states should be examined and whether they should be analyzed independently (as single-items) or aggregated to emotional-state scales. This variability can cause methodological challenges since emotions differ in both valence (negative vs. positive) and arousal levels (high vs. low), impacting metabolic processes and appetitive responses [35]. Thus, instead of aggregating emotion items to arbitrary scales, this study explored the factor structure of the emotion items accounting for valence and arousal [36–39].

### Food craving as a shared antecedent of eating behavior across patients with different eating disorders and individuals without eating disorder

Despite existing studies on emotional eating across some eating disorders [e.g., 40, 41], no study has applied a unified measure to compare food craving in patients with AN-R, AN-BP, BN, BED, and control individuals, addressing a significant gap in understanding universal eating behavior predictors. Thus, food craving was chosen as an outcome measure as it is a common predictor and mediator of various overt eating behaviors [42, 43]: increased food craving could predict binge eating, while decreased food craving could predict restriction. The validity of food cravings in emotional eating studies is further supported by laboratory studies and food craving diaries in patients with BN and BED [44–46]. This level of abstraction is necessary as, studying emotional eating across eating disorders and in healthy individuals on the same variable precludes measuring overt eating behaviors that are only shown by patients with certain eating disorders and that are not seen in healthy individuals at all (i.e., severe restriction and binge eating).

### Aims and hypotheses

Under the affect regulation model, negative emotions and higher food cravings (as proxy for binge eating) were expected to co-occur in patients with binge-type eating disorders [BN and BED; i.e., 2, 24], and negative emotions and lower food cravings (as proxy of restriction) were expected to co-occur in patients with AN-R [31] relative to individuals in the CG. Due to the mixed symptomology in AN-BP (i.e., restriction, binge eating, and purging) their emotion-food-craving patterns might take an intermediate position between the patients with AN-R and those with BN [17, 47].

Further, positive emotional states may foster functional eating patterns in patients with eating disorders as reported empirically [4, 10, 11, 16] and as suggested by the broaden-and-build theory [13, 15]. Thus, positive emotions and lower food cravings were expected to co-occur in patients with binge-type eating disorders (i.e., less dysfunctional eating behavior), while positive emotions and higher food cravings were expected to co-occur in patients with AN-R, relative to individuals from the CG [4].

The study also explores group differences in food cravings as a function of emotional states with different valence (negative vs. positive) and arousal levels (high vs. low). Negative emotions with high arousal (i.e., anger and frustration) and higher food cravings were expected to co-occur in patients with BN. Negative emotions with low arousal (i.e., depressiveness) and higher food cravings were expected to co-occur in patients with BED, relative to individuals from the CG. Such eating disorder specific associations of binge eating and high vs. low arousal negative emotions have been found by Castellini et al. [34].

## Methods

### Participants

Most patients with eating disorders (*n* = 83) were recruited from the waiting list for inpatient treatment at a large clinic in southern Germany specialized on eating disorder treatment. Additional patients (*n* = 36) and individuals in the CG were recruited from the general public. The ethics committees of the University of Salzburg and the University of Munich approved the study, and all participants signed an informed consent form.

In total, 204 participants were categorized into the following diagnostic groups: CG *n* = 58, AN-R *n* = 29, AN-BP* n* = 26, BN *n* = 50 and BED *n* = 41. All patients fulfilled diagnostic criteria for current AN-R, AN-BP, BN or BED from the diagnostic and statistical manual of mental disorders-5 [DSM-5; 48]. Exclusion criteria were ‘eating disorders otherwise specified’, ‘eating disorders not otherwise specified’, and positive screening for psychosis. Exclusion criteria for the CG were current or lifetime eating disorders, a body mass index < 18.5 kg/m^2^ or  ≥ 25.0 kg/m^2^. All participants were ≥ 14 years old and of female sex. The sample comprised university students (38.7%), employees (31.9%) and other occupations (29.4%). As expected, groups differed regarding body mass index (BED > BN > CG > AN-BP and AN-R). Patients with BED were older than the participants in the other groups (see Table 1).

**Table 1:** Sociodemographic data and trait self-report measures per group

	*M* (*SD*, *range*)				
	CG^A^	AN-R^B^	AN-BP^C^	BN^D^	BED^E^
EMA compliance	83.1 (12.9, 50–100)	76.3 (15.3, 52.1–95.8)	82.5 (12.3, 52.1–100)	78.4 (14.8, 50.0–100)	82.1 (10.9, 62.5–97.9)
BMI (kg/m^2^)	20.7 (1.5, 18.6–24.5)^B,C,D,E^	15.6 (1.9, 11.6–18.1)^ A,D,E^	16.4 (1.4, 13.0–18.4)^ A,D,E^	23.1 (3.1, 19.0–29.7)^ A,B,C,E^	31.5 (7.6, 18.9–46.3)^ A,B,C,D^
Age	24.2 (8.3, 16–53)^ E^	26.8 (12.9, 14–60)^ E^	25.7 (11.4, 16–57)^ E^	29.3 (10.4, 16–53)^ E^	36.6 (11.6, 18–59)^ A,B,C,D^
Years of education	14.7 (2.5, 11–22)	13.5 (5.4, 9–38)	13.6 (3.3, 7–19)	14.9 (3.9, 10–26)	15.1 (4.7, 3–27)
EDEQ	1.3 (1.0, 0.2–4.4)^ B,C,D,E^	3.1 (1.0, 1.0–5.6)^ A,C,D^	4.5 (0.8, 2.7–5.8)^ A,B,E^	4.3 (0.9, 1.5–6.0)^ A,B,E^	3.1 (1.1, 0.3–5.5)^ A,C,D^
CES-D	34.9 (7.3, 23–53)^ B,C,D^	44.4 (9.7, 28–62)^ A^	46.3 (8.4, 28–66)^ A,E^	43,6 (11.4, 25–66)^ A^	38.4 (10.3. 23–65)^ C^
FCQ-T-r	37.5 (14.2, 15–81) ^C,D,E^	35.4 (12.7, 15–67)^ C,D,E^	57.5 (17.6, 27–88)^ A,B,D^	69.2 (11.4, 49–90)^ A.B,C,(E)^	60.4 (13.4, 17–82)^ A,B,(D)^
SEES happiness	3.1 (0.4, 2–4)^ D,E^	3.3 (0.5, 2–3) ^D,E^	2.8 (0.7, 2–4)^ E^	2.7 (0.9, 1–5)^ A,B^	2.3 (0.7, 1–4)^ A,B,C^
SEES sadness	3.3 (0.7, 2–5)^ B,D,E^	2.3 (0.7, 1–4)^ A,C,D,E^	3.0 (1.2, 1–5)^ B,D,E^	4.0 (0.8, 2–5)^ A,B,C^	4.2 (0.6, 2–5)^ A,B,C^
SEES anger	2.7 (0.6, 1–4)^ D,E^	2.2 (0.7, 1–4)^ D,E^	2.4 (1.0, 1–5)^ D,E^	3.3 (0.8, 2–5)^ A,B,C^	3.5 (0.8, 1–5)^ A,B,C^
SEES anxiety	2.5 (0.7, 1–5) ^D,E^	2.1 (0.5, 1–3)^ D,E^	2.6 (1.0, 1–5)^ E^	3.2 (0.8, 1–5)^ A,B^	3.3 (1.1, 1–5)^ A,B,C^
DEBQ emo	31.9 (9.9, 14–61)^ B,D,E^	23.1 (11.5, 13–51)^ A,C,D,E^	34.2 (13.7, 14–63)^ B,D,E^	49.1 (10.8, 23–65)^ A,B,C^	49.0 (8.2, 14–64)^ A,B,C^

Bold, upper-case letters indicate significant Scheffé tests respective to the following groups: ^A^CG = comparison group (without eating disorders and with normal weight); ^B^AN-R = anorexia nervosa restrictive subtype; ^C^AN-BP = anorexia nervosa binge-purge subtype; ^D^BN = bulimia nervosa; ^E^BED = binge-eating disorder. EMA = ecological momentary assessment. BMI = body mass index. EDEQ = Eating Disorder Examination Questionnaire [64, German version: 65]. CES-D = Center for Epidemiologic Studies Depression Scale [66, German version: 67]. FCQ-T-r = Food Craving Questionnaire-Trait reduced [68, German version: 69]. SEES = Salzburg Emotional Eating Scale [German version: 70]. DEBQ = Dutch Eating Behaviour Questionnaire [71, German version: 72]

### Measures

#### Clinical diagnostic interviews

The Structured Clinical Interview for DSM-IV [SCID; 49] and the Eating Disorder Examination [EDE; 50] were conducted. Both interviews were adapted to DSM-5 diagnostic criteria. This was necessary as no DSM-5 conform interviews were available in German at the beginning of data acquisition (January 2018–May 2021). Trained, female, master-level students, graduate students, and post-doctoral researchers conducted the diagnostic interviews.

#### Sociodemographic measures and trait questionnaires

Participants reported sociodemographic data (i.e., age, weight, height, years of education) and questionnaire data (i.e., eating disorder pathology, depressiveness, trait food craving, emotional eating) via an online questionnaire.

#### EMA measures

Participants received six EMA signals (9 am, 11.30 am, 2 pm, 4.30 pm, 7 pm, and 9.30 pm) per day for 8 days, each probing momentary emotions and momentary food craving, as well as other variables that are not relevant here. Responses could be delayed for up to one hour, thereafter EMA signals expired.

##### Momentary food craving

Participants were asked to rate their momentary food craving on a continuous rating slider (“Do you have a desire to eat something tasty right now?”; from *not at all* [0], to *very much* [100]). Thus, the intensity of momentary food craving was measured by assessing its ‘desire to eat’ component [51].

##### Momentary emotional states

Participants were asked to rate their momentary emotional state on continuous rating sliders (“How are you feeling right now?” *irritable, worried, nervous or stressed, tense, depressed, bored, dissatisfied with myself, relaxed, calm, cheerful,* and *enthusiastic*; from *not at all* [0], to *very much* [100]). The 11 emotion items were presented in randomized order.

#### Procedure

At the start of the study, all participants signed the informed consent form and completed an online questionnaire about sociodemographic data and trait measures. Participants underwent the clinical diagnostic interview (~ 1-2 h) on the phone and were instructed on how to install and use the customized EMA application *PsyDiary*. The day of the installation was not used in the analyses and served to familiarize the user with the app. Participants responded to six signal-contingent EMA questionnaires per day for 8 days. Parts of the present sample participated in a larger project (for a list of all related and unrelated papers on parts of this project, see AppendixA). All participants received personalized feedback on their EMA data and either 1–2 course credits (for psychology students at the University of Salzburg) or €10–25–depending on their EMA response rate.

### Statistical analyses

#### Multilevel confirmatory factor analyses of emotion items

Aggregation of EMA emotion items [i.e., from the Positive and Negative Affect Scale, PANAS; 52] to positive and negative emotion scales is often criticized for overtly reducing important facets of emotions [i.e., arousal; 36–39]. Therefore EMA emotion items were grouped empirically on distinct scales by performing several multilevel confirmatory factor analyses using the R package lavaan [53]. Considering literature [36–39] five different factor solutions were optimized and tested against each other.

The data were nested (level1–observations in level2–participants). Thus, a random intercept effect was specified for participants. The models were estimated with robust maximum likelihood. Several analyses tested the impact of lower loading items on the psychometric properties of the scales and compared all solutions. The Chi-square test, the comparative fit index (CFI), the Tucker–Lewis index (TLI), the root-mean-square error of approximation (RMSEA), and the standardized root mean residual (SRMR) were used as main test values [54–56]. See AppendixB for details.

#### Group differences in the association of momentary emotional states and food craving

To investigate the main hypotheses, a multilevel modeling approach was used. All multilevel models were set up with nested random effect structure [57]. Rstudio [58] and the package lme4 [59] were used to calculate the multilevel models. ‘Momentary food craving’ was modeled as a dependent variable. The four continuous ‘momentary emotional-state scales’ (as determined by the multilevel confirmatory factor analyses) were person-mean centered with the R package misty [60] and modeled as level1 predictors, in four separate models. ‘Group’ was modeled as a categorical level2 predictor. Multilevel interactions between ‘momentary emotional-state scales’ and ‘group’ were added to assess group-specific associations of emotions and food craving. In a forward model selection process, random intercepts (for participants) and random slopes (for emotional states) were tested in addition to the fixed effects (emotional states, groups and their multilevel interaction). The final models were controlled for overly influential cases by excluding participants with values over four times the mean Cook’s distance with the R package Influence ME [61]. The R package lmerTest [62] was then used to examine the results of the multilevel models: Omnibus tests were used to test for significant interactions between each emotional-state scale and group. Significant interactions were followed up with pairwise comparisons of the simple slopes of each possible pair of groups. Code and data are available at the Open Science Framework [63]. See AppendixC for a priori power analyses.

## Results

In total 7904 (of 9792 possible) EMA signals were answered before the timeout of the respective questionnaire and thus analyzed. On average 38.75 of 48 EMA signals were answered per participant, representing a compliance of 80.73% (*SD* = 13.42, *range* 50–100%). Groups did not differ in their compliance with the EMA protocol (see Table 1).

### Multilevel confirmatory factor analyses of emotion items

Threshold values for goodness-of-fit indices were met (i.e., CFI > 0.95 and TLI > 0.90 [73], RMSEA < 0.08 [55], SRMR < 0.08 [56]). Regarding the Chi-square test, the lowest possible value was looked for [73, 74].

The final best fitting model (see model^d^; Table 2) included all 11 original emotion items on the following four latent factors and outperformed several other factor solutions: (1) negative valence high arousal (Neg_High_): *irritable, nervous-stressed, tense*; (2) negative valence low arousal (Neg_Low_): *depressed, bored, worried, dissatisfied with myself*; (3) positive valence low arousal (Pos_Low_): *relaxed, calm*; (4) positive valence high arousal (Pos_High_): *cheerful, enthusiastic*. The covariances are not fixed to 0 as fixing them worsened the model fit (cf. model^**d**^ and model^**e**^; Table 2).

**Table 2:** Results of multilevel confirmatory factor analyses on the emotion items

Model	χ^2^	*df*	CFI	TLI	RMSEA [90% CI]	SRMR Level 1	SRMR Level 2	AIC	BIC	SABIC
One factor^a^ (emotions)	6539.1	88	0.766	0.707	0.096 [0.093, 0.098]	0.074	0.166	750,568.7	750,952.3	750,777.5
Two factors^b^ (arousal)	3748.8	86	0.772	0.708	0.096 [0.093, 0.098]	0.074	0.174	750,405.8	750,803.3	750,622.2
Two factors (valence)^c^	2461.3	86	0.866	0.828	0.073 [0.071, 0.076]	0.060	0.087	747,831.0	748,228.6	748,047.5
**Four factors (corr)**^**d**^	**1123.3**	**76**	**0.963**	**0.946**	**0.041 [0.038, 0.044]**	**0.035**	**0.078**	**745,176.9**	**745,644.2**	**745,431.3**
Four factors (uncorr.)^e^	5648.9	88	0.641	0.551	0.119 [0.116, 0.121]	0.244	0.401	753,990.0	754,373.6	754,198.8

Used observations (level 1) *n* = 7904, used clusters (level2) *n* = 204. *CFI *comparative fit index, *TLI* Tucker–Lewis index, *RMSEA*  root-mean-square error of approximation, *CI* confidence interval, *SRMR* standardized root mean residual. The four-factor model fitted significantly better compared to all other models with *p* < 0.001. Fixing covariances of the latent factors in models with multiple latent factors consistently worsened model fits this is exemplified with the comparison of model^c^ and^d^. See Appendix B for other disregarded factor solutions

Mean scores were calculated for each scale. McDonald’s omega (*ω*) coefficients –as estimates of model-based reliability–were satisfactory to good (level1: ω_NegHigh_ = 0.76, ω_NegLow_ = 0.59, ω_PosHigh_ = 0.81, ω_PosLow_ = 0.76; level2: ω_NegHigh_ = 0.84, ω_NegLow_ = 0.82, ω_PosHigh_ = 0.88, ω_PosLow_ = 0.82). Table3 reports group means of the resulting four EMA scales (Neg_High_, Neg_Low_, Pos_High_, Pos_Low_) as well as food craving. Compared to CG participants, individuals in the eating disorder groups reported higher negative emotions and lower positive emotions, except for individuals with BED.

**Table 3:** Means and standard deviations of averaged momentary emotional states and momentary food craving per group

	*M*, (*SD*)				
	CG^A^	AN-R^B^	AN-BP^C^	BN^D^	BED^E^
Neg_High_ (irritable, nervous-stressed, tense)	18.9 (15.8) ^B,C,D^	30.6 (22.7) ^A^	36.9 (23.2) ^A,E^	34.5 (24.4) ^A,E^	25.7 (20.8) ^C,D^
Neg_Low_ (depressed, bored, worried, dissatisfied with myself)	14.7 (12.5) ^B,C,D,E^	30.5 (19.9) ^A^	40.6 (21.0) ^A,E^	38.0 (21.1) ^A,E^	24.3 (18.3) ^A,C,D^
Pos_High_ (relaxed, calm)	26.7 (22.6) ^B,C,D,(E)^	18.8 (21.1) ^A^	14.1 (17.2) ^A^	17.2 (19.7) ^A^	19.8 (22.2) ^(A)^
Pos_Low_ (cheerful, enthusiastic)	37.9 (26.7) ^B,C,D^	25.0 (26.2) ^A^	19.7 (21.1) ^A,(E)^	23.8 (24.1) ^A^	28.9 (26.6) ^(C)^
Food craving	42.9 (28.6) ^B^	28.3 (25.0) ^A,C,D,E^	44.0 (30.7) ^B^	48.7 (31.2) ^B^	48.0 (32.1) ^B^

*Neg*_*High*_ negative valence-high arousal; *Neg*_*Low*_ negative valence-low arousal; *Pos*_*High*_ positive valence-high arousal, *Pos*_*Low*_ positive valence-low arousal. Bold, upper-case letters indicate significant Scheffé tests for mean differences to the following groups: ^A^*CG *comparison group (without eating disorders and with normal weight); ^B^*AN-R* anorexia nervosa restrictive subtype; ^C^AN-*BP* anorexia nervosa binge-purge subtype; ^D^*BN* bulimia nervosa; ^E^*BED*  binge-eating disorder

### Group-specific associations of emotions with food craving

**Fig. 1:**
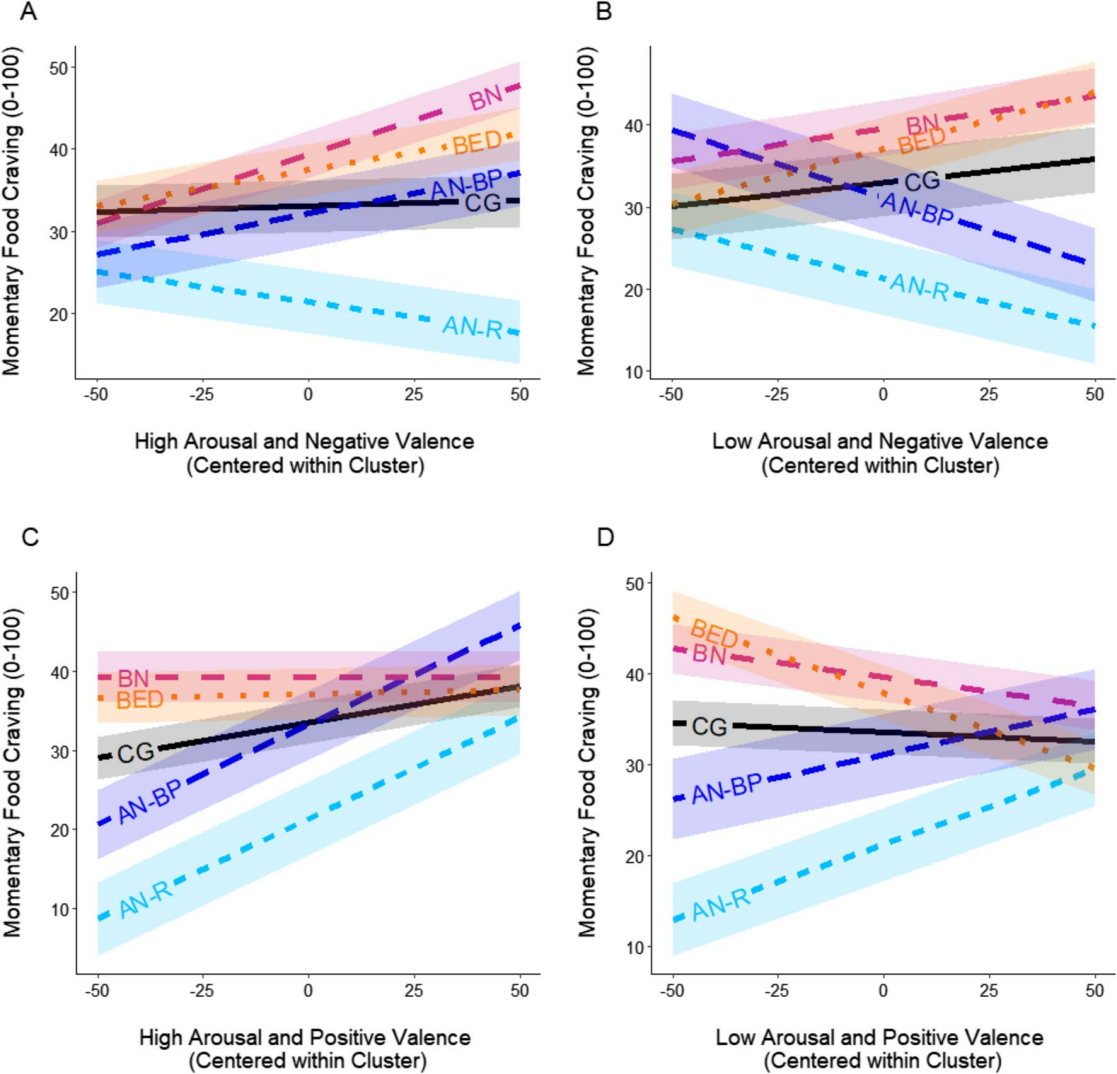
Visualization of simple slopes for momentary emotional states predicting food craving for each group. Groups: *CG* comparison group (without eating disorders and with normal weight), *AN-R* anorexia nervosa restrictive subtype, *AN-BP* anorexia nervosa binge-purge subtype, *BN* bulimia nervosa, *BED* binge-eating disorder. Each panel shows the simple slope for one emotional-state scale: **A** high arousal-negative valence, **B** low arousal-negative valence, **C** high arousal-positive valence, **D** low arousal-positive valence

Regarding the four multilevel models testing the main hypotheses about group-specific associations of emotions and food craving: Omnibus tests indicated interactions between ‘group’ and each of the four ‘emotion scales’ (all *p*’s ≤ 002). This justified follow-up pairwise comparisons of the simple slopes of food craving predicted seperatly by each of the four* ‘*emotion scales*’* (Neg_High_, Neg_Low_, Pos_High_ and Pos_Low_; i.e., negative or positive valence and high or low arousal) of all groups against each other (CG, AN-R, AN-BP, BN, BED). The results from these pairwise comparisons can be found in Table 4 and Fig. 1.

**Table 4:** Pairwise comparisons of simple slopes for ‘momentary food craving’ predicted by ‘momentary emotions’, ‘group’ and their interactions

Pairwise comparisons of simple slopes *β* (*SE*)				
Compared groups	Model with high arousal negative valence	Model with low arousal negative valence	Model with high arousal positive valence	Model with low arousal positive valence
CG vs. AN-R	0.09 (0.07)	0.18 (0.10) †	− 0.16 (0.08) *	− 0.19 (0.06) **
CG vs. AN-BP	− 0.09 (0.07)	0.22 (0.09) *	− 0.16 (0.07) *	− 0.12 (0.07) †
CG vs. BN	− 0.16 (0.06) *	−0.02 (0.09)	0.09 (0.06)	0.04 (0.04)
CG vs. BED	− 0.08 (0.06)	− 0.08 (0.09)	0.08 (0.05)	0.15 (0.04) ***
AN-R vs. AN-BP	− 0.17 (0.07) *	0.05 (0.10)	0.00 (0.10)	0.07 (0.08)
AN-R vs. BN	− 0.24 (0.06) ***	− 0.20 (0.09) *	0.26 (0.09) **	0.23 (0.07) ***
AN-R vs. BED	− 0.16 (0.07) *	− 0.25 (0.09) **	0.24 (0.08) **	0.33 (0.06) ***
AN-BP vs. BN	− 0.07 (0.06)	− 0.25 (0.08) **	0.25 (0.08) ***	0.16 (0.07) *
AN-BP vs BED	0.01 (0.07)	− 0.30 (0.09) ***	0.24 (0.07) **	0.26 (0.07) ***
BN vs. BED	0.08 (0.06)	− 0.05 (0.08)	− 0.01 (0.06)	0.10 (0.05) *
Conditional pseudo-R^2^	0.24	0.24	0.25	0.25

Groups: CG = comparison group (without eating disorders and with normal weight), *AN****-****R* anorexia nervosa restrictive subtype, AN-*BP* anorexia nervosa binge-purge subtype, *BN* bulimia nervosa, *BED* binge-eating disorder

Significance codes: *** = *p* < 0.001; ** = *p* < 0.01; * = *p* < 0.05; † = *p* < 0.10

## Discussion

The present study is the first to examine the association of empirically grouped emotional states with food craving in everyday life in patients with AN-R, AN-BP, BN and BED compared to individuals without eating disorders and with normal weight. Unique emotional food craving patterns in each group were found: Negative emotions covaried with dysfunctional patterns of food craving (i.e., *higher* food craving in the BN group and *lower* food craving in both AN groups), while positive emotions covaried with food craving patterns that approach ‘healthy’ food craving levels of the CG (*higher* food craving in both AN groups and *lower* food craving in the BED group).

### Negative emotions and food craving

In *AN-BP (*and in *AN-R* at trend level*)*, compared to the CG, elevated Neg_Low_ emotions and lower food craving co-occurred. This is in line with qualitative and questionnaire research in AN [4, 75–77]. Interestingly, when testing the slopes of *AN-BP* and *AN-R,* opposing patterns were revealed during Neg_High_ (*higher* food craving in AN-BP, *lower* food craving in AN-R). Thus, in *AN-R*, negative emotional states show a more generalized hypo-appetite link to food craving. In AN-BP (compared to AN-R), the direction of that link is more arousal-dependent, which could underlie the mixed symptom presentation in AN-BP (i.e., restriction, binge-eating, and purging). This difference further supports the distinction between the two subtypes as emphasized in other research as well [4, 17, 78, 79].

In *BN*, elevated Neg_High_ emotions and higher food craving co-occurred, a pattern that was not seen in individuals of the CG. This is in line with findings on emotion-related binge eating [e.g., 80, 81]. Similar results were also obtained in a food-craving-diary study and a laboratory food-cue-reactivity experiment [44, 46].

The *BED* group did not differ from the CG regarding negative emotion-related food craving*,* unlike EMA studies on emotion-related binge-eating would suggest [e.g., 2, 34]. The BED sample, however, might have been less pathological compared to previous studies (i.e., no differences between BED and CG on trait depressiveness and Neg_High_, Pos_High_ and Pos_Low_; see Tables 1, 3).

### Positive emotions and food craving

In *AN-R* and *AN-BP*, compared to the CG, evidence for higher food craving during elevated positive emotions was found (irrespective of emotional arousal). This could be considered a healthy and functional response in underweight patients as it might encourage food intake. In *BED* lower food craving during elevated Pos_Low_ was found—again, a potentially healthy response in a group with frequent overweight. From this angle, food craving levels in AN-R, AN-BP and BED seem to ‘normalize’ (approaching CG food craving levels) during higher intensities of positive emotional states (see Fig. 1C–D). This is in line with evidence describing healthier eating behavior during positive emotional states in clinical samples [4, 10, 11]. That said, some literature suggests the contrary, i.e., that positive emotional states can also trigger unhealthy eating behaviors [18–21]. This might explain why no such normalization was observed in the *BN* group.

### Dimensional emotion models in emotional eating theories

The high specificity of emotion-food-craving associations in each eating disorder group in the present results clearly contrasts with a uniform ‘negative-overeating’ construct (i.e., generally increased appetitive responses during negative emotions). The results suggest that the emotion-food-craving associations depend on a) eating disorder (sub)group, b) valence (positive vs. negative), and c) arousal (high vs. low) of the co-occurring emotional state. These differentiated patterns may explain some of the inconsistencies in literature when either emotional states of varying arousal or different eating disorder (sub-)groups are combined, as frequently done in experimental and EMA studies [e.g., 24, 82].

On a very general level, the results suggest that several mechanisms might be at play across eating disorders. This heterogeneity would explain the multitude of emotional eating theories. The findings specifically support the value of the *affect regulation model* to explain ‘negative emotional overeating’ in BN and ‘negative emotional undereating’ in AN-BP (and AN-R). The *broaden-and-build theory* may further explain healthier eating patterns during positive emotions in both AN groups and in BED: The repertoire of accessible cognitions and actions is broadened and thus, may help the patients to think of the bigger picture and act out healthier behaviors [16].

### Strengths and limitations

The present study comprised a large sample (*N* = 204 participants and *N* = 7904 EMA observations), including female patients with AN-R, AN-BP, BN, BED, and a large female CG. Statistical reliability of emotion scales was assured by means of multilevel confirmatory factor analysis. The multilevel modeling approach further assured adequate accounting of the full within- and between-person variance of the EMA data. These statistical and methodological points support the internal and external validity of the findings.

Yet, as indicated above, the results rest on the assumption that food craving is a valid proxy for subsequent food intake. While there is good evidence in that regard [42, 43, 83–87] more research on actual binge-eating episodes and restriction is needed. Further, as the sample comprises female individuals only, the findings cannot be generalized to male individuals. The present study focused on female participants because they tend to show more emotional (binge) eating compared to males [88], and because of concern about not finding an adequate proportion of male patients for all eating disorder subgroups in a reasonable period. Therefore, replications with male participants are warranted.

### What is already known on this subject?

While negative emotions are often related to maladaptive (disordered) eating behaviors, there is equivocality regarding which specific negative emotions may drive emotional over-/undereating in different eating disorders [4, 34]. Thus, the present study aimed to close two gaps: (1) grouping emotions on emperical dimensions that relate to different eating behaviors (e.g., valence and arousal dimensions of emotions [36–39]). (2) Assessing emotions and food cravings across different eating disorders.

### What this study adds?

The present study illustrates that emotions and food craving are uniquely associated for each eating disorder. However, emotional valence and arousal need to be considered to find these unique associations. Hence, the present results can guide subgroup-specific theorizing and targeted interventions on both binge eating (in binge-type eating disorders) and restriction (in restrictive-type eating disorders): a traditional ‘problem-centered’ approach would focus on the reduction of negative emotions, addressing both hyper-appetitive (i.e., increased food craving in BN during Neg_High_ states) and hypo-appetitive eating behaviors (i.e., depressed food craving in AN-BP and AN-R during Neg_Low_ states). The results on positive emotions also command to a salutogenetic approach of eating disorder treatment that focuses on increasing positive emotions [89], particularly regarding the reduction of binge-eating urges in BED and stimulation of appetitive behaviors in AN-R [cf., 10, 16]. As for novel interventions, emotions with specific valence and arousal levels could be detected in real-time during daily life [90] to trigger context-appropriate interventions [91], i.e., to stimulate appetitive behaviors or prevent binge eating.

## Data Availability

Data and R code are available at the Open Science Framework [https://osf.io/euzm3/].
